# From the Western Alps across Central Europe: Postglacial recolonisation of the tufa stream specialist *Rhyacophila pubescens *(Insecta, Trichoptera)

**DOI:** 10.1186/1742-9994-8-10

**Published:** 2011-05-15

**Authors:** Christine HM Engelhardt, Peter Haase, Steffen U Pauls

**Affiliations:** 1 Senckenberg, Department of Limnology and Conservation, Clamecystr. 12, 63571, Gelnhausen, Germany; 2 Biodiversity and Climate Research Centre (BiK-F) Senckenberganlage 25, D-60325 Frankfurt am Main, Germany; 3 University of Minnesota, Department of Entomology, 219 Hodson Hall, 1980 Folwell Ave, Saint Paul, MN 55108, USA

## Abstract

**Background:**

Dispersal rates, i.e. the effective number of dispersing individuals per unit time, are the product of dispersal capacity, i.e. a species physiological potential for dispersal, dispersal behaviour, i.e. the decision to leave a habitat patch in favour of another, and connectivity of occupied habitat. Thus, dispersal of species that are highly specialised to a certain habitat is limited by habitat availability. Species inhabiting very stable environments may also adopt a sedentary life-style. Both factors should lead to strong genetic differentiation in highly specialised species inhabiting stable environments. These two factors apply to our model species *Rhyacophila pubescens *a highly specialised freshwater insect that occurs in tufa springs, a very stable habitat.

**Results:**

We examined the genetic population structure and phylogeography using range-wide mtCOI sequence and AFLP data from 333 individuals of *R. pubescens*. We inferred the location of Pleistocene refugia and postglacial colonisation routes of *R. pubescens*, and examined ongoing local differentiation. Our results indicate intraregional differentiation with a high number of locally endemic haplotypes, that we attributed to habitat specificity and low dispersal rates of *R. pubescens*. We observed high levels of genetic diversity south of the Alps and genetic impoverishment north of the Alps. Estimates of migrants placed the refugium and the source of the colonisation in the Dauphiné Alps (SW Alps).

**Conclusions:**

This is the first example of an aquatic insect with a colonisation route along the western margin of the Alps to the Central European highlands. The study also shows that specialisation to a stable environment may have promoted a behavioural shift to decreased dispersal rates, leading to stronger local population differentiation than in less specialised aquatic insects. Alternatively, the occurrence of highly specialised tufa spring habitats may have been more widespread in the past, leading to range regression and fragmentation among present day *R. pubescens *populations.

## Background

In recent years our knowledge of phylogeographic patterns of European animal and plant species has grown tremendously [1-4]. From these studies we are gaining a better understanding of the biogeography of the European flora and fauna and how current species distribution patterns were shaped by both ancient and recent earth history [5]. We have also learned that terrestrial species may exhibit different patterns than aquatic species [6-8]. Species of aquatic insects, in particular, can show different patterns of population structure, even if they are co-distributed, are closely related, and/or share the same ecological niche [9,10]. Historic population movement and changes in effective population size, but also recent or ongoing gene flow among populations, shape present-day patterns of population structure. Current dispersal rates and gene flow result from the dispersal capacity of a species (i.e. its physiological ability to disperse and successfully find and recolonize new habitats), its dispersal behaviour [11], and the connectivity of suitable habitats. The latter can be reduced if species are highly specialised in their habitat requirements [11,12].

Compared to terrestrial niches, stream biotopes are erratically distributed, making their inhabitants particularly interesting for studying population genetics and phylogeography. Many highland aquatic insect species exhibit 'insular' distributions among mountain ranges, but also within mountain ranges where populations occur in isolated habitats with few or no interconnecting corridors of suitable habitat. This is due to the linear structure of stream habitats and the habitat specificity of many species [13], the restriction of lateral dispersal to the generally short-lived, winged adult stage, and the behaviour of species to disperse primarily along stream corridors [14,15]. Some aquatic insects, particularly some species of caddisflies, are considered good fliers, and long-distance dispersal has been documented for several species [16-19]. Thus, dispersal behaviour may play a very prevalent role in shaping genetic population structure in aquatic insects [20]. Species living in rare, isolated, but more or less stable habitats are expected to disperse less frequently than species living in ephemeral or more common habitats [19-22]. Permanent springs and spring brooks are particularly stable as their physical-chemical parameters, e.g. temperature, are less prone to seasonal or annual variation [23,24]. Tufa springs, defined as calcareous springs with calcium carbonate deposits, have constantly high pH and conductivity [25]. Thus, it is perceivable that tufa spring specialist aquatic insect species may exhibit particularly low dispersal rates, independent of adult dispersal ability.

The caddisfly *Rhyacophila pubescens *Pictet, 1834, is a highly specialised cold-stenotherm species that only occurs in permanent tufa spring brooks in limestone mountain ranges from the spring source to 5 km downstream [26,27]. Using mitochondrial COI sequence data, we previously examined the population structure of *R. pubescens *north of the Alps [28] and observed one central haplotype in all regions north of the Alps. This haplotype putatively gave rise to many other haplotypes that differed from it by one or two mutations. Based on this pattern we hypothesized that *R. pubescens *postglacially recolonised its Central European range from a single refugial source. We also observed numerous private haplotypes and hypothesized recent or ongoing *in-situ *diversification. Since our sampling was limited to the northern ranges and excluded common European refugial areas, and we examined only mtDNA sequences, we could not test these hypotheses in our previous study.

Our current study has three main objectives. First, we explicitly test the hypothesis of a postglacial colonisation of Central Europe from a single Pleistocene refuge. We predict that genetic data will show a connection between one, not several, southern refugia and the Central European populations. Second, we want to identify the location of the Pleistocene refugia of *R. pubescens*, which we predict to be associated with the south-western Alps based on calcareous Pleistocene refugia known from the region for plants [29] or with refugia on the Italian Peninsula [1-3,5]. Upon identifying the refugia we wish to reconstruct the population history and recolonisation process of *R. pubescens*. Third, we ask if highly specialised inhabitants of stable environments - in this case *R. pubescens *- exhibit lower dispersal rates and higher levels of population differentiation than other species that are less specialised. We predict that *R. pubescens *exhibits high levels of population structure, even at a small geographic scale, due to its strong affiliation with isolated tufa spring environments. To address these objectives we use a range-wide sampling of both mtDNA sequence and nuclear Amplified Fragment Length Polymorphisms (AFLP) data. We employ both population genetic and phylogeographic methods to elucidate patterns of population differentiation, past migration rates and changes in demographic history.

## Materials and methods

**Figure 1:**
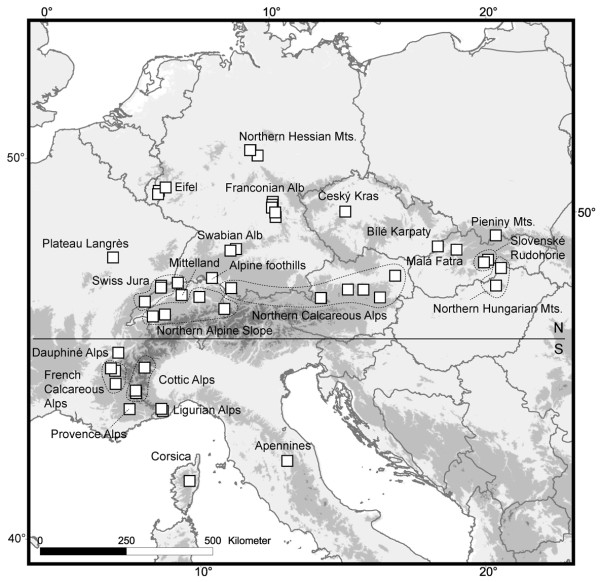
**Map of sampling sites covering the entire distribution range of *R. pubescens***. Mountain regions are named according to Table 1. Horizontal line shows regions north and south of the Alps as referred to in this study. The Map was produced in ESRI GIS based on GTOPO30. "Mts." = Mountains.

We analysed 333 specimens of *R. pubescens *from 51 sites across the entire distribution range (Figure 1, Table 1). MtCOI sequence data for 197 specimens from the northern distribution were taken from Engelhardt et al. [28]; additional sequences from the remaining distribution area and all AFLP data were newly generated. Collection and storage of specimens followed Engelhardt et al. [28]. Larvae and adults were identified using Waringer & Graf [30] and Malicky [31], respectively. All specimen vouchers are deposited at Senckenberg, Germany.

**Table 1:** Sampling sites of *R. pubescens *listed by mountain ranges (ranges are separated by horizontal lines).

Mountain region	**Country**^**#**^	Number of individuals for mtCOI/AFLP	Nr. of endemic haplotypes/mountain region	Stream name, locality^*^	Latitude (°N)	Longitude (°E)	Collector	Haplotype (number of individuals)^*^
Northern Hessian Mts. (HE)	DE	5/3		Flachsbach above Wendershausen	51.30167	9.88778	Engelhardt & Hövelborn	H1(5)
		7/4		Gatterbach above Wanfried	51.18306	10.22639	Engelhardt & Hövelborn	H2(7)
		2/2	0	**Griesbach**	51.30278	9.87583	Engelhardt & Hövelborn	**H1(2)**

Franconian Alb (FRA)	DE	6/5		Burglesauer Bächlein above Burglesau	49.99611	11.08722	Engelhardt	H1(6)
		8/4		Tributary Ellerbach above Tiefenellern	49.91667	11.07972	Engelhardt	H1(5), H3(3)
		7/6		Brook below Tiefenhöchstädt	49.84111	11.07611	Engelhardt	H1(3), H4(3), H5(1)
		7/1		Rüsselbach at Kirchrüsselbach	49.60139	11.27167	Engelhardt	H1(4), H6(2), H7(1)
		8/5	4	Hundshauptener Bach below Hundshaupten	49.72139	11.23028	Engelhardt	H1(5), H2(3)

Swabian Alb (SWA)	DE	3/3		Attenriedbach, Geislingen	48.62139	9.81639	Mayer	H1(2), H2(1)
		8/2	0	Fils above Wiesensteig	48.55944	9.59889	Engelhardt & Schlünder	H1(8)

Eifel (EI)	DE	8/7		Hygropetric, Tränenlay	49.85500	6.32361	Engelhardt, Pauls & Neu	H1(8)
	LU	5/4		Spring near Haalerbach	49.76667	6.31667	Graf	H2(5)
	LU	4/4	1	Walpengraben near Metterich	49.98222	6.58111	Bálint & Neu	H1(2), H2(1), H58(1)

Northern Calcareous Alps (NCA)	AT	8/7		Brook near Möggers	47.56167	9.81694	Graf	H1(8)
		1/1		Bertaquelle, Hollensteingraben	47.66778	15.76139	Graf	H1(1)
		2/2		Schreiberbach, Wiener Wald	48.27417	16.33444	Graf & Pauls	H1(2)
								
		9/7		Mayrgraben, Lunz	47.85000	15.08333	Malicky	H1(9)
		1/1		Weißenbach, Reichraming	47.83111	14.46139	Graf	H1(1)
		3/3		**Teufelsgraben**	47.54528	13.41944	Pauls & Theissinger	**H29(2), H30(1)**
		1/1	2	**Brook above Dygrub**	47.55139	13.41389	Engelhardt	**H1(1)**

Alpine foothills (AFO)	DE	6/4	2	Mühltalbach above Möggingen	47.76250	9.00806	Sundermann	H8(4), H9(2)

Mittelland (ML)	CH	6/4	1	Talbach above Pratteln	47.50528	7.68611	Engelhardt & Lehrian	H1(2), H10(2), H11(1), H12(1)

Swiss Jura (JU)	CH	8/7		**La Motte above Ocourt**	47.35000	7.05667	Engelhardt & Lehrian	H1(3), H13(2), H14(1), H24(1), **H59(1)**
		8/5		Dénériax, Noirvaux	46.85722	6.51722	Engelhardt & Lehrian	H1(3), H10(4), H18(1)
		8/6		Brook above Soubey	47.30250	7.05861	Engelhardt & Lehrian	H1(6), H21(1), H25(1)
		1/0	6	**Chrintelbachquellen**	47.43083	7.88361	Pauls	**H1(1)**

Northern Alpine slope (NAS)	CH	5/5		Nameless brook, Bächenmoos	47.20861	8.61306	Vicentini	H1(5)
		6/5		Nameless brook, Prantin	46.49694	6.92417	Engelhardt & Lehrian	H1(2), H19(4)
		4/4		Warmbach above Weissenbach	46.60056	7.37833	Engelhardt & Lehrian	H1(3), H20(1)
		8/6	3	Brook near Fanas	46.98139	9.66111	Lubini	H1(7), H22(1)

Pieniny Mts. (PIE)	PL	2/2	0	Pieninski Potok	49.41611	20.39889	Szczesny	H1(2)

Bílé Karpaty (BK)	CZ	3/3	1	Tributary of Kloboucký Potok	49.10250	18.01833	Chvojka	H27(3)

Český Kras (CK)	CZ	6/6	1	Císařská rokle SW of Srbsko	49.91806	14.13333	Engelhardt & Schlünder	H26(6)

Mala Fatrá (MFA)	SK	8/7	0	Valcansky Potok, Martin	49.02278	18.78389	Engelhardt & Bieber	H1(8)

Slovenské Rudohorie (SLR)	SK	8/4		Biele Vody, Murán	48.76000	20.07694	Engelhardt, Blanár & Trebulová	H1(6), H4(1), H28(1)
		7/4	3	Potok Kamenárka, Tisovec	48.69028	19.91111	Engelhardt, Blanár & Trebulová	H15(6), H23(1)

Northern Hungarian Mts. (HU)	HU	6/6		Tributary, Menes Völgy, Aggtelek	48.54083	20.59806	Engelhardt & Bieber	H2(4), H16(2)
		6/3	2	Ban, Bükk Mountains	48.06750	20.39444	Kiss	H1(5), H17(1)

Plateau Langrès (PLA)	FR	16/14	2	**Cascade d'Etuf**	47.87500	4.96528	Engelhardt & Kind	**H1(2),H13(1), H31(11), H32(2)**

Dauphiné Alps (DA)	FR	12/4	2	**Nameless brook near Les Miards**	44.88722	5.85167	Engelhardt & Kind	**H1(1), H13(8), H33(1), H34(2)**

French Calcareous Alps (FCA)	FR	7/4		**Lalley**	44.92361	5.67472	Bálint	**H1(4), H2(2), H12(1)**
		4/3		**Torrent de la Sapie**	44.53833	5.95083	Engelhardt & Kind	**H1(1), H35(2), H36(1)**
		17/13	2	**Saint-Philibert, Grande Chartreuse**	45.37972	5.84917	Bálint	**H1(13), H2(2), H56(2)**

Cottic Alps (CA)	FR	5/5		**Jausiers**	44.39000	6.77600	Bálint	**H35(4), H52(1)**
	FR	5/3		**La Condamine-Châtelard**	44.45100	6.74100	Bálint	**H35(5)**
	IT	6/6	2	**Tributary of Dora Riparia**	45.10000	6.93333	Engelhardt & Kind	**H45(2), H46(4)**

Provence Alps (PA)	FR	18/15	3	**Ravin de Chambiéres**	43.93278	6.63694	Engelhardt & Kind	**H1(3), H37(4), H38(10), H39(1)**

Ligurian Alps (LA)	IT	12/8		**Nameless brook near Rezzo**	44.02583	7.86667	Engelhardt & Kind	**H46(3), H47(1), H48(1), H49(1), H50(1), H51(2), H53(1), H54(1),H57(1)**
		8/8	9	**Valle di Pietra**	44.07722	7.80639	Delmastro	**H55(8)**

Apennines (APP)	IT	7/7	3	**Tributary of Fiume Tescio**	43.09722	12.67556	Engelhardt & Lehrian	**H42(1), H43(3), H44(3)**

Corsica (COR)	FR	7/7	2	**Tributary of Tavignano**	42.25639	9.20583	Engelhardt & Kind	**H40(3), H41(4)**

^#^Country codes according to ISO 3166. *Mt DNA sequences generated for this study are in bold print, others were taken from Engelhardt et al. [28].

### Molecular Methods

#### Mitochondrial sequence data

DNA extraction and PCR amplification protocols of a 475bp long fragment of mtCOI followed Engelhardt et al. [28]. Sequences were generated by Nano+Bio Center Kaiserslautern, Germany, and AGOWA GmbH Berlin, Germany. ABI traces were aligned, checked, and edited manually using Sequencher Vers. 4.8 (Gene Codes Corporation, Michigan, USA).

#### Amplified Fragment Length Polymorphism

The Amplified Fragment Length Polymorphism (AFLP) protocol followed Huck et al. [32] with minor modifications: Genomic DNA concentration was standardised to 50 ng DNA/μl. 250 ng genomic DNA were digested. The initial restriction-ligation lasted 14 h at 20°C. Multiplex AFLP products were genotyped on an ABI Prism 3100 DNA capillary sequencer (University of Mainz, Germany) together with an internal size standard (GeneScan ROX 500, ABI). Fragments were scored with Genemarker Vers. 1.7 (SoftGenetics, Pennsylvania, USA). Fragments were automatically scored as present when peak height exceeded the standard parameter-setting threshold (300). Trace files were also re-examined visually. Fragments in the size range of 100-250 bp were used for analysis. We used 18 replicate samples to assess scoring error according to Bonin et al. [33]. Twelve fragments were not included in the analyses because of scoring errors; 19 fragments were only present in one or two individuals and were thus excluded; one monomorphic fragment was also excluded.

### Analyses

#### Mitochondrial sequence data

To examine genetic population structure we pooled the 51 sampled sites by mountain region and uniform geological units following Diercke Weltatlas [34] and Gonseth et al. [35]. This grouping is non-random but reflects the existing geographic isolation of *R. pubescens *across the distribution range. Samples were grouped into 23 different geographic units, i.e., mountain regions, which we refer to as regions (Table 1). We calculated an unrooted median-joining haplotype network [36] in Network 4.5.0.1 (Fluxus Technology) to illustrate haplotype distribution. Exact tests of population differentiation [37] and pairwise *F*_ST_-values were used to detect differentiation among regions. We partitioned total genetic variation by geographic hierarchies using Analysis of Molecular Variance (AMOVA) [38]: geographic hierarchies were "among 23 regions", "among populations, i.e. sampling sites, within regions" and "within populations". We also calculated independent AMOVAs for the regions north and south of the Alps (Figure 1). A Mantel test [39] for isolation-by-distance was conducted using pairwise *F*_ST_-values and geographical distance between all analysed populations. Analyses were performed with Arlequin 3.1 [40] using default settings, except for the AMOVAs that were run with 16,000 permutations. Mismatch distributions were calculated with 1,000 bootstraps for 23 regions and for the whole dataset. To test for demographic change in each region and the whole dataset, we calculated two neutrality tests: Tajima's *D *and Fu's *F*_*S*_. Significant negative *D *and *F*_*S *_values can arise under selective effects but can also indicate population expansion or bottlenecks [41,42]. We also calculated gene and nucleotide diversity. Analyses were performed in Arlequin 3.1 with default settings.

#### Amplified Fragment Length Polymorphism

A Mantel test [39] was conducted using pairwise *F*_ST_-values and geographical distance between all analysed populations with 999 permutations in GenAlEx 6.1 [43]. Using AFLPdat [44] we calculated the proportion of polymorphic markers (95% confidence) and Nei's gene diversity H [45] in each region and for the regions north and south of the Alps. We also calculated frequency down weighted marker values DW for each region [46]. High DW-values would be expected in older populations where rare markers should accumulate due to mutations, low values are expected in recently established populations. Following Westberg & Kadereit [47] we used AFLPdiv [48] with rarefaction set to 4, to assess intrapopulation genetic diversity as band richness (br_4_), i.e. "the number of phenotypes expected at each AFLP locus when four individuals have been sampled from the population." Due to limited sample size we did not evaluate br_4 _in the Bilé Karpaty and Pieniny Mountains. The Shannon Index of phenotypic diversity S [49] was calculated in POPGENE 3.2. Private fragments, i.e. fragments that only occured in one region or stream population were counted using AFLPdat, to assess the degree of divergence among populations and regions [50]. AMOVA [38] was calculated for the AFLP data in Arlequin 3.1 with 16,000 permutations.

We selected the regions where we sampled three or more streams (Northern Hessian Mountains, Franconian Alb, Northern Calcareous Alps, Swiss Jura and Northern Alpine slope) to assess ongoing or recent diversification among populations within regions. We calculated mean GST [45] among populations within these regions using POPGENE 3.2 [51].

#### Model-based coalescent estimates of migration

The distribution of the ancestral haplotype H1 in the northern populations and the Western Alps (see Results), combined with the strongly diverged and differentiated haplogroups associated with the Ligurian Alps and the Apennines, indicates that the most probable location of the refuge and source for the northward colonisation is somewhere in the Western Alps. We therefore used Migrate 3.0.3 [52] to investigate past gene flow in the Western Alps region to examine from where the putative northward colonisation originated. We calculated a stepping stone model for the populations Provence Alps, French Calcareous Alps, Dauphiné Alps, Swiss Jura, Mittelland and Northern Alpine slope (Table 1) to estimate effective migrants. The model was set with asymmetric migration parameters and unrestricted theta estimates. Thus the model estimated migration between neighbouring populations. Starting values were estimated from *F*_ST_-values for the first run. We conducted a second run using the estimates for theta and M of the first run as starting values. Both runs used 10 short chains and recorded 25,000 genealogies with a sampling increment of 20 (500,000 genealogies visited), and two long chains that recorded 200,000 genealogies with as sampling increment of 50 (10,000,000 genealogies visited). We used an adaptive heating scheme with four chains (1.0, 1.5, 2.5, 5.0) and a swapping interval of one to ensure sufficient mixing. The analysis was based on the mtCOI dataset.

## Results

### MtDNA haplotype distribution

**Figure 2:**
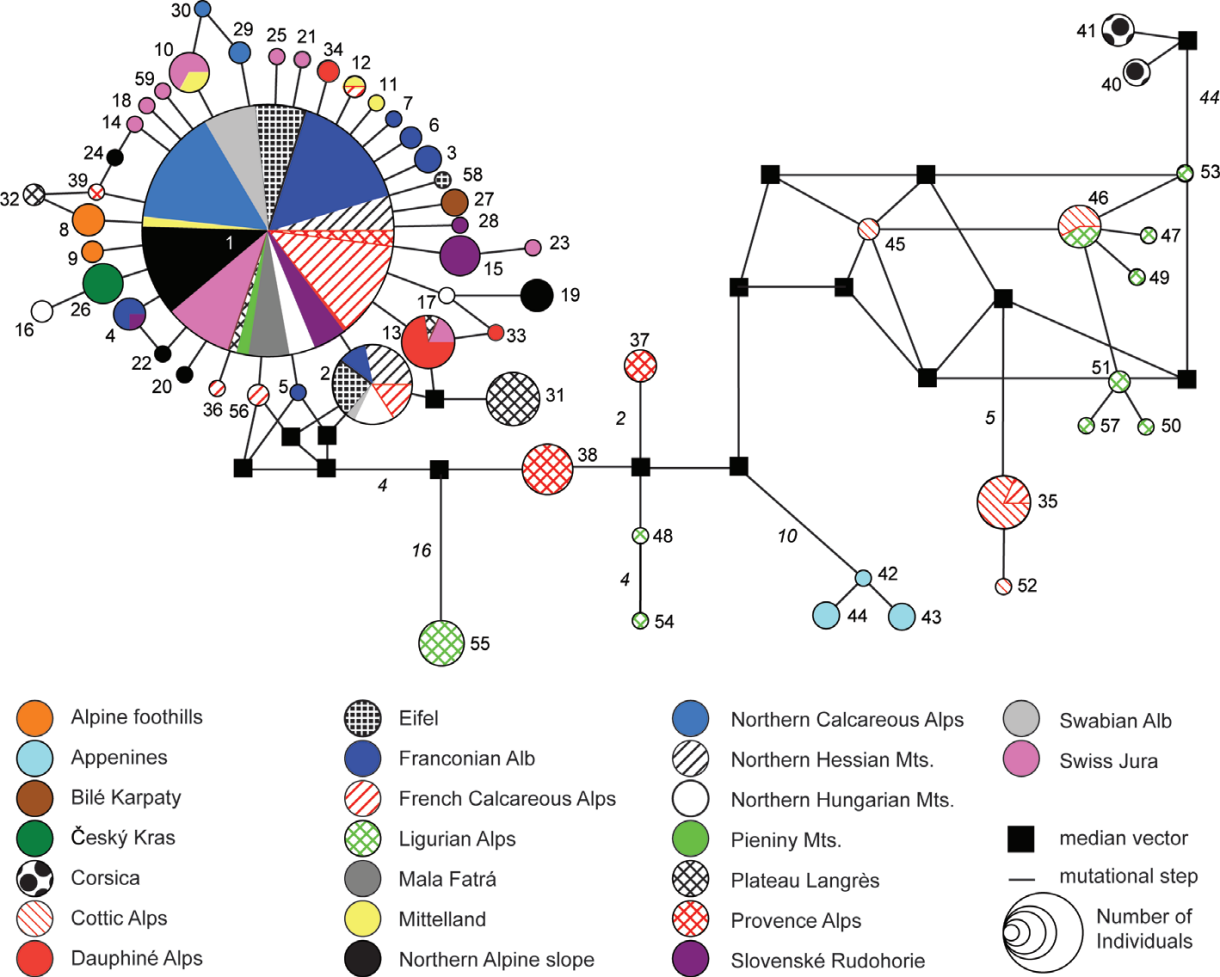
**Median-joining haplotype network of *R. pubescens *(mtCOI)**. Colours code for mountain regions. Size of haplotypes is relative to the number of individuals carrying this haplotype. Numbers code for haplotypes, numbers in bold italics indicate mutational steps > 1. "Mts." = Mountains.

We generated unambiguous mtCOI sequences for 333 individuals. The 475 bp alignment contained no gaps or length variants, 94 positions were variable and 83 sites were parsimony informative. There were 59 unique haplotypes (GenBank Accessions EU885387-EU885414, GU186972-GU187002). The maximum difference was 70 bp (14.74%) between all haplotypes and 29 bp (6.11%) for the "Central European" haplotypes without the divergent haplotypes found in Liguria, the Apennines and Corsica. The unrooted median-joining haplotype network (Figure 2) showed that the northern populations were dominated by one central haplotype, H1, which was carried by almost half of the specimens examined (N = 149). This central haplotype was surrounded by several haplotypes that differed from it by one or two mutational steps. H1 was not present in the Northern Alpine foothills, the Český Kras and the Bilé Karpaty, the Cottic and Ligurian Alps, the Apennines or on Corsica. In all regions north and south of the Alps there were regional endemic haplotypes and haplotypes endemic to single streams (Table 1). In the Western Alps, Ligurian Alps, Apennines and on Corsica haplotypes were highly divergent. A Bayesian Markov-Chain Monte Carlo phylogenetic inference (results not shown) was consistent with the median-joining network.

### Genetic diversity

**Figure 3:**
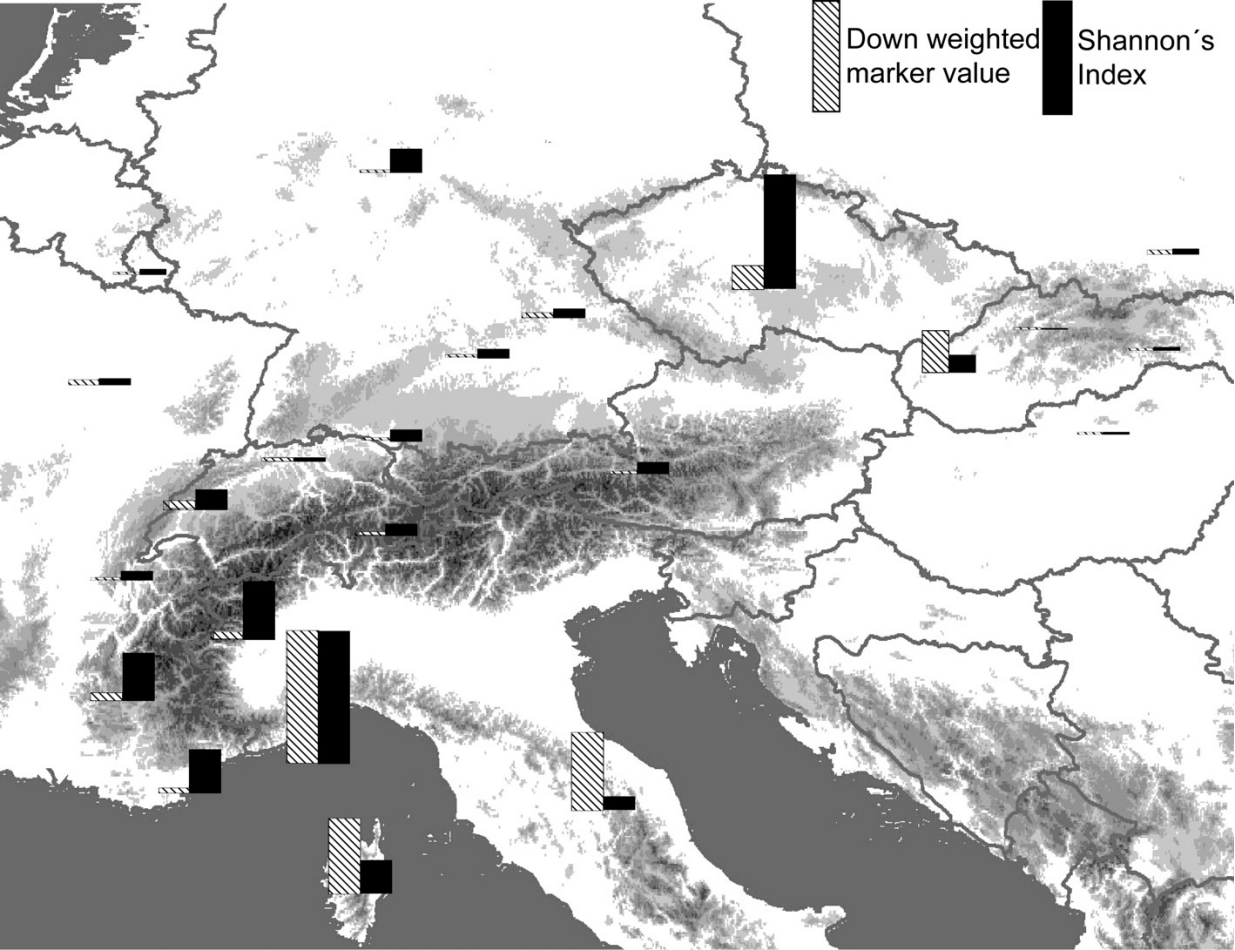
**Shannon's index and down-weighted marker value for AFLP samples for each region**. Heights of bars indicate relative values compared to the highest value found for each index.

The final AFLP dataset comprised 132 fragments. Maximum scoring error at individual loci was 0.11; mean mismatch value per fragment over all samples was 0.05. Gene diversity based on mtCOI data of northern populations was 0.7290 +/- 0.0289, and 0.9240 +/- 0.0134 for southern populations. Nucleotide diversity was also lower in the north (0.012868 +/- 0.009120) than in the south (0.161948 +/- 0.080769). The percentage of polymorphic AFLP loci (95% confidence), band richness and Nei's gene diversities based on AFLPs were highest in the Ligurian Alps, followed by the Česky Kras, Cottic Alps, French Calcareous Alps and the Provence Alps (Table 2). Percentage of polymorphic loci was lower in the region north of the Alps (0.63%) than in the region south of the 231 Alps (0.95%), as was Nei's gene diversity (north: 0.05, south: 0.21). Shannon Index of phenotypic diversity based on AFLP was highest in the Western Alps, on Corsica and in the two populations from the Czech Republic (Table 2, Figure 3). As a measure of divergence the frequency down-weighted marker value (DW) was calculated for AFLPs. We found the highest value in Liguria, and high values in the Apennines and on Corsica, and in the Czech populations (Table 2, Figure 3). Private AFLP fragments were present in the Apennines (4 fragments), Corsica (4), Liguria (19), the Cottic Alps (1), and the Franconian Alb (1). Fixed private fragments (i.e. private fragments that occur in all sampled individuals from the respective population) were found in the Apennines (1) and on Corsica (3).

**Table 2:** Genetic diversity estimators of *R. pubescens *populations detected by AFLP's.

Mountain Region *	Prop. of polymorphic loci	**Band richness (br**_**4**_)**	Nei's gene diversity H	Shannon Index	DW-value from means
HE	0.14	1.080	0.043	0.06	57.98
FRA	0.05	1.027	0.015	0.022	98.4
SWA	0.05	1.038	0.02	0.024	46.91
EI	0.04	1.021	0.011	0.017	45.31
NCA	0.11	1.033	0.017	0.03	53.41
AFO	0.05	1.053	0.028	0.031	59.22
ML	0.02	1.015	0.008	0.009	77.59
JU	0.11	1.062	0.034	0.05	178.34
NAS	0.09	1.032	0.017	0.028	77.2
PIE	0.02	n.c.	0.023	0.016	103.11
BK	0.08	n.c.	0.056	0.053	943.3
CK	0.48	1.416	0.233	0.283	457.82
MFA	0.01	1.004	0.002	0.003	39.12
SLR	0.02	1.014	0.007	0.01	39.74
HU	0.02	1.010	0.005	0.008	40.19
PLA	0.05	1.018	0.009	0.015	110.81
DA	0.04	1.038	0.02	0.022	39.65
FCA	0.3	1.145	0.078	0.12	144.69
CA	0.3	1.188	0.104	0.147	151.61
PA	0.21	1.138	0.076	0.107	93.13
LA	0.58	1.428	0.241	0.33	2504.97
APP	0.05	1.046	0.026	0.032	1484.31
COR	0.15	1.114	0.063	0.081	1423.44

*Letters indicate mountain regions according to Table 1.

** "n.c.": not calculated

### Population differentiation and genetic structure

Exact tests of population differentiation based on mtCOI data indicated that 214 of 253 (84.6%) of all region pairs were significantly differentiated (see Additional File 1). Pairwise *F*_ST_-values were significant for 182 of 253 comparisons (71.9%, p ≤ 0.05, Bonferroni adjusted α-value = 0.00020, see Additional File 1). Pairwise *F*_ST _values of AFLP data were significant for 123 of 253 comparisons (48.62%, p ≤ 0.05, Bonferroni adjusted α-value = 0.00020, see Additional File 2).

AMOVA of both mtDNA and AFLP data showed that populations in different regions were genetically different from each other (mtCOI: 73.06%; AFLPs: 57.53%; both p < 0.001) and that variance among populations within regions was much lower (mtCOI: 26.94%; AFLPs: 42.47%; both p < 0.001). When only taking the mountain ranges north of the Alps into account, AMOVA of mtCOI data revealed more variation within regions (63.78%, p < 0.001) than among regions (36.22%, p < 0.001). In contrast, AMOVA of AFLPs from the northern populations showed 45.72% variation within regions and 54.28% variation among regions (p < 0.001 for both values). In the southern regions there was less variation within regions (mtCOI: 24.95%; AFLPs: 46.46%; both p < 0.001) than among regions (mtCOI: 75.05%; AFLPs: 53.54%; both p < 0.001). The results illustrate that differentiation among mountain ranges north of the Alps was lower than among mountain ranges south of the Alps. In general, the differences were less pronounced in the AFLP data than in the mtCOI data. A weak isolation-by-distance effect was revealed by Mantel test (mtCOI: r = 0.151759, p = 0.02; AFLP: r = 0.226, p < 0.01). A Mantel test considering only the populations north of the Alps showed no correlation based on mtCOI data (r = 0.038254, p = 0.30), but did show a weak correlation in the AFLP data (r = 0.244, p < 0.01). In both data sets a much stronger correlation was found in the southern populations (mtCOI: r = 0.434078, p < 0.01; AFLP: r = 0.666, p < 0.01), indicating that the southern populations were comparatively closer to equilibrium between genetic drift and gene flow than the northern ones.

We examined ongoing or recent diversification using GST in the Northern Hessian Mountains, Franconian Alb, Northern Calcareous Alps, Swiss Jura and Northern Alpine slope. Mean GST among populations within each of the five regions was 0.54 in the Northern Calcareous Alps, 0.49 in the Swiss Jura, 0.45 in the Northern Alpine slope, 0.41 in the Northern Hessian Mountains and 0.14 in the Franconian Alb.

### Demographic expansion

In the regions studied north of the Alps, almost all mismatch distributions of mtCOI haplotypes were unimodal [28]. Unimodal distribution of pairwise differences indicates recent population growth [53]. In the southern regions most of the mismatch distributions were bi- or multimodal except for the Dauphiné Alps and the Apennines, indicating stable population sizes in the south without any hint of population expansion. Negative significant values for Tajima's *D *were found for the Swiss Jura, the French Calcareous Alps and for the dataset as a whole, indicating a high number of low frequency polymorphisms in the mtCOI dataset and potential population size expansion [41,42] (results not shown). Values of Fu's *F*_*S *_test for mtCOI data were negative and significant for the Franconian Alb and the entire dataset, and highly significant for the Swiss Jura (results not shown). Negative, but non significant values for both tests were found in the Swabian Alb, Northern Calcareous Alps, Mittelland, Slovenské Rudohorie, Northern Hungarian Mountains and the Dauphiné Alps.

### Migration in the Western Alps

**Figure 4:**
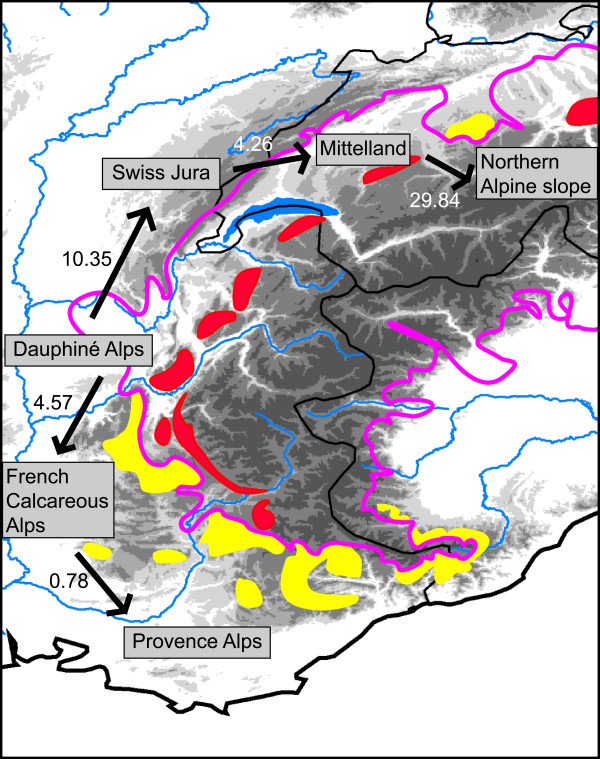
**Relative migration rate values (N_m) between each population pair for the stepping stone model for the Western Alps region (mtCOI data)**. Arrows show inferred direction of effective migration. Pink line shows the maximum glacial expansion 20000 years bp; coloured areas represent inferred refugia on calcareous bedrock (yellow: refugia outside the LGM glacial extension; red: potential nunatak refugia below the permanent snowline, but within the maximum glacial expansion). Maximum extent of glaciation and inferred refugia were redrawn from Schönswetter et al. [29].

We used Migrate to test the hypothesis that the refuge and source for the northern populations was located in the south-western (SW) Alps. We calculated a stepping stone model with mtCOI data to estimate numbers of effective migrants and the direction of migration from the populations in the SW Alps to the northern populations. Both Migrate runs yielded similar results. Results of the second run are presented. Gene flow, measured as effective migrants, was detected from the Dauphiné Alps southward to the French Calcareous Alps, and from these to the Provence Alps (Figure 4). There was also northward gene flow from the Dauphiné Alps to the Swiss Jura, to a higher degree from the Swiss Jura to the Mittelland and from there to the Northern Alpine slope. No gene flow was detected from the Provence or Calcareous Alps northward or from the Swiss Jura southward.

## Discussion

### Glacial refugia and postglacial recolonisation of *R. pubescens*

#### Glacial Refugia

*Rhyacophila pubescens *is restricted to Central Europe and the Italian peninsula, the latter a region of many Pleistocene refuges [1,5]. Circum-alpine refugia are also postulated for several aquatic insects [8,54,55]. Accordingly, we consider the Apennines or the south-western Alps - the latter also on the basis of haplotype sharing with all central European populations [28] - as possible refugial zones. We can dismiss the Apennines as a likely source of refuges for the northern populations, because the genetic composition of these populations differs dramatically from those of Central European populations. Instead, our data show that the location of the refugium was in the western part of the Alps. This is supported by the fact that haplotype H1, which is the ancestral haplotype of the northern populations, is not present in the Italian Peninsula or Liguria, but in the French part of the Alps. Also, all other haplotypes in Central Europe are derived from H1, showing that the genetic make up of the Central European populations was primarily influenced from a common refugial source.

We thus propose that the northern edge of the distribution of *R. pubescens *during the last glacial maximum was in the region of the French Calcareous or Dauphiné Alps, below the permafrost line and that northward expansion started from there. The results of gene flow analysis indicate that the Dauphiné Alps are the only region from which migration occurred northwards and southwards in the Western Alps (Figure 4). The northward migration presumably coincided with gradual climate warming in the early Holocene, about 10,000 years ago [56]. It would seem plausible that the French and Swiss Jura were recolonised first, as the glacial retreat was slower in the higher regions of the main Alpine ridge. Gene flow and migration rates indicate a recolonisation route along the Western Alps to Switzerland and then to the Central European highlands.

Colonisation from the southwestern Alps seems plausible since potential peripheral refugia with calcareous bedrock have also been inferred for mountain plants [29]. A southwestern Alps refugium and subsequent recolonisation from there was shown for the plant *Eryngium alpinum *that also exhibits a strong affinity to calcareous substrate [57]. A northward recolonisation route along the Western Alps was also inferred for the butterfly *Polyommatus coridon*, a species typical of calcareous grasslands [58]. Interestingly, a glacial tongue was present near the present-day city of Gap during the last glacial maximum that could have caused a period of separation between the Liguria/Provence populations and the French Calcareous Alps/Dauphiné Alps populations [29]. When this glacial tongue retreated, gene flow occurred again between the French Calcareous Alps/Dauphiné Alps and the Provence Alps. This scenario is concordant with the results of gene flow analysis in this study and would explain the finding of both the "northern" haplotype H1 and the"southern" haplotypes H37, H38 in the Provence Alps. Based on haplotype distribution and results of the Migrate analysis, we infer a secondary contact zone for *R. pubescens *in the Provence Alps.

Overall, haplotype divergence, molecular variance and genetic diversity in *R. pubescens *are much greater in the southern part of the range than in the north. Increasing genetic impoverishment from former refugia to recently recolonised areas is an expected and common pattern in organisms [59-61], including aquatic species like the bryozoan *Cristatella mucedo *[62], and the gastropod *Theodoxus fluviatilis *[63]. The latter species exhibits low genetic diversity in mtDNA in populations in Northern Europe, where all haplotypes seem to be derived from a single ancestral haplotype, similar to the pattern we observed in *R. pubescens*. High values of these diversity estimators are generally expected in populations that are relatively old or in hybrid zones. Diversity indices and down-weighted marker values derived from AFLP data indicate that the south-western Alps, the Apennines and Corsica have been inhabited continuously by *R. pubescens*. Results from mtDNA and AFLP analyses both support present complete isolation of the Corsican populations. In the haplotype network (Figure 2) H53 from Liguria is the closest haplotype to the Corsican cluster, and is separated from it by 45 mutational steps (9.47% of 475 bp). This degree of divergence is evidence of long-term isolation of the Corsican lineage from the remaining mainland populations. Monophyly of all known *R. pubescens *haplotypes from the entire distribution was confirmed by a three gene phylogeny of six closely related species in the *Rhyacophila tristis*-group [64], but it seems evident that the Corsican population of *R. pubescens *is in the process of speciation. Clarifying the divergence times of the Corsican population in the context of a phylogenetic study of the *R. tristis*-group is a logical next step. While promising interesting biogeographical findings with regard to Corsican Trichoptera, a detailed examination is not central to our research questions and exceeds the scope of our current study.

#### Postglacial colonisation of Central Europe

During the early Holocene (~10,000 years ago), vast areas of Central Europe were covered by thick loess deposits [65]. It is known that tufa formation occurred in these loess deposits [66], though the exact processes are not yet fully understood. It seems reasonable to assume that this period was characterised by a highly variable climate and dynamic fluvial processes [67], which may have forced recolonising species like *R. pubescens *to disperse to more moderate environments or more stable streams. The cold-tolerance of the species and its ability to cope physiologically and functionally with very high carbonate concentrations could have promoted rapid recolonisation of Central Europe. Our records [27,28,64] illustrate that the species is able to inhabit calc-sinter streams where other macroinvertebrate predators are very rare. Thus, the species appears to be currently outcompeted in less marginal habitats by other macroinvertebrate predator species. However, *R. pubescens' *physiological plasticity should be tested in laboratory experiments.

More frequent long distance dispersal or more widespread suitable habitat during this period of recolonisation would explain the presence of the common haplotype H1 over the entire northern part of the present range of *R. pubescens*. Other examples of rapid northward recolonisation were shown to occur in the pond turtle *Emys orbicularis *[67], and even in flightless species like the grasshopper *Chorthipppus parallelus *[60].

*Rhyacophila pubescens *is the first example of a Central European aquatic insect that started postglacial recolonisation from a south-western alpine refugium along the western edge of the Alps to the former periglacial area north of the Alps. This pattern differs considerably from patterns of Pleistocene survival and postglacial recolonisation of Central Europe observed in other cold tolerant caddisflies and aquatic invertebrates, for example, multiple glacial refugia (*R. aquitanica *[68], *D. romanicus *[10]) or Central European refugia (e.g. *D. discolor *[8]).

The case study in *R. pubescens *provides another example that phylogeographic history appears to be largely species-specific in aquatic insects with no common patterns emerging to date. This is quite different to the situation in terrestrial species, where several common patterns are known [3,5]. Differences among terrestrial and aquatic responses to historic climate change may result from the fundamental difference in thermal regimes of terrestrial and aquatic ecoystems, and stream ecosystems in particular. Specific differences observed among stream dwelling aquatic insects likely relate to the different habitat specialisation of the species, their cold-tolerance, their dispersal ability, habitat availability during major glaciations and in the postglacial period of recolonisation or a combination of these factors.

### Habitat specialisation, population differentiation, and dispersal behavior

The caddisfly *R. pubescens *is adapted to a specialised habitat that also happens to be characterised by stable environmental conditions: small permanent headwater tufa streams and springs. While we sampled a variety of habitat types from all regions over several years, *R. pubescens *was only collected from tufa springs, underscoring its restriction to this habitat type. As is to be expected for any species that occurs in patchily distributed headwater stream environments with specific physico-chemical characteristics, *R. pubescens *exhibits regional differentiation. A similar pattern was also observed  in other highland caddisflies in Europe [8,10,68]. However, even within regions where there were no obvious barriers to dispersal between suitable habitats, we detected genetic differentiation in *R. pubescens*, as evidenced by GST and private haplotypes. The presence of private haplotypes in almost all mountain ranges and in single streams across the entire distribution of the species indicates low dispersal rates between streams. This observation is supported by the absence of an isolation-by-distance effect in the northern populations. The lack of an isolation-by-distance pattern across much of the northern part of the range of *R. pubescens *indicates that higher genetic drift in marginal populations is not the main reason for the observed pattern [69]. *R. pubescens *is rarely collected in large numbers [27], and low effective population sizes combined with low or zero gene flow between habitat patches, in addition to genetic drift, may be changing the composition of each local gene pool [70,71].

Several studies suggest that highly specialised species are more isolated because of habitat availability than generalist species. A comparatively lower local dispersal rate may result in a high number of rare or locally restricted alleles as shown for the butterfly *M. aurelia *[72], a calcareous grassland specialist. Matern et al. [12] also inferred a low dispersal capacity and a high degree of within drainage genetic differentiation for the headwater specialist beetle *Carabus variolosus*. Molecular studies of European caddisflies have not yet examined differences among specialist and generalist species, but the available studies do allow for some comparisons. In *Hydropsyche tenuis *the genetic diversity and differentiation are lower than in *R. pubescens *and there is evidence for ongoing or recent long-distance dispersal surrounding the Alps in *H. tenuis *[9]. *Hydropsyche tenuis *is less selective regarding its habitat, occurring in the very dynamic headwater and mid-range stream regions of calcareous and siliceous streams [13]. *Rhyacophila aquitanica*, *Drusus discolor*, and *Drusus romanicus *are more strongly associated with cold habitats than *H. tenuis*, but occur in both siliceous and calcareous streams. They exhibit greater population differentiation than *H. tenuis *[8,10,68], but the degree of intraregional differentiation is not nearly as pronounced as it is in *R. pubescens*. The comparison with the available studies does suggest that specialisation may lead to greater population differentiation in aquatic insects.

Why is *R. pubescens *apparently not dispersing among its specialised habitats? At first, this result seems at odds with the widespread occurrence of the dominant haplotype and the hypothesis of a rapid postglacial recolonisation of Central Europe. The observed patterns are consistent with a recent reduction of suitable habitat and resulting local allopatric fragmentation. The vast loess deposits over Central Europe in the early Holocene [65] may have provided more widespread tufa habitats [66] suitable for *R. pubescens*. After the postglacial expansion and the decrease in loess deposits across Europe, the primarily sedentary species would have experienced allopatric fragmentation into more isolated outcrops of calcareous rocks where tufa streams form and persist to the present. Unfortunately with so little knowledge about tufa development in loess deposits and limited paleosol data, it is currently not possible to reconstruct the distribution of tufa habitats during the early Holocene to test this idea.

The contrast between widespread haplotypes and many private fragments and haplotypes is also consistent with a shift in dispersal rates either following a change in dispersal capacity of the species, or a change in dispersal behavior. We favour the explanation of a shift in dispersal behaviour over a relatively recently evolved morphological or physiological adaptation resulting in reduced dispersal capacity. Hoffsten [73] showed that morphology of the thorax and its effect on flight was linked to site occupancy in many stream species of caddisflies. In his study he examined two species of *Rhyacophila*, both of which showed morphological attributes related to strong flight (e.g., relatively high relative thoracic mass, which reflects the amount of flight muscle available). However one species, *R. fasciata *was limited in the number of sites where it occurred (27%), while *R. nubila *occurred at all sites (100%). Thus, while both species have the physiological capacity for flight and dispersal, one species disperses less. Dispersal rates are not limited solely by flight capacity, i.e. the physiological ability to fly certain distances or for certain periods of time, but also by behaviour. If dispersal behaviour is linked to habitat stability [19-22], then it seems reasonable that *R. pubescens*, which currently only inhabits environmentally stable tufa springs and headwater streams, has adopted a more or less sedentary life style with little lateral dispersal among streams. The widespread occurrence of a common haplotype suggests that long distance dispersal is possible in *R. pubescens*, but in this specialist caddisfly, dispersal rate may be responding to habitat availability and habitat persistence [21]. Current geographic conditions, the stability of spring habitats, and the high degree of habitat specialisation of *R. pubescens *could be promoting a predominantly sedentary behaviour.

## Conclusions

Our study shows that changes in habitat availability through time or plasticity in ecological life history traits can shape a species' distribution pattern and genetic population structure. This is particularly true for high specialised species. In response to inhabiting a very stable but generally harsh environment (tufa springs), *R. pubescens *may have adopted a more or less sedentary behavior with limited dispersal rates leading to rare exchange of genetic material among populations and thus the evolution of locally restricted haplotypes and AFLP fragments. Nevertheless diversity indices and shallow genetic population structure show that widespread postglacial dispersal from a southern refuge and occupancy of habitats north of the Alps was possible, highlighting the species' high physiological dispersal capacity. This apparent contradiction suggests a shift in dispersal behaviour or availability of habitat between the early postglacial and today. Our study species shows Pleistocene persistence and postglacial colonisation from a single refugial source in the southwestern Alps, a pattern hitherto unknown in aquatic insects. Both aspects highlight the specificity of aquatic species responses to past and potentially future climate change.

## Competing interests

The authors declare that they have no competing interests.

## Authors' contributions

CHME, PH, and SUP conceived and designed the study. CHME carried out the molecular genetic studies. CHME and SUP analyzed the data and wrote the manuscript. All authors read and approved the final manuscript.

## Supplementary Material

Additional file 1 Regional differentiation based on mtCOI data. Click here for file

Additional file 2 Regional differentiation based on AFLP data (*F*_ST_). Click here for file
