# Supplementary material for: From the Western Alps across Central Europe: Postglacial recolonisation of the tufa stream specialist Rhyacophila pubescens (Insecta, Trichoptera)
Source: Front Zool. 2011 May 15;8:10. doi: 10.1186/1742-9994-8-10 (PMC3119172; doi:10.1186/1742-9994-8-10)
Supplement: Additional file 1 — Regional differentiation based on mtCOI data. [file 1742-9994-8-10-S1.DOC]

Additional File, Engelhardt et al FIZ, EngelhardtEtAlFIZ_TableA1.doc

Table A1: Regional differentiation based on mtCOI data. Results of exact tests of population differentiation (Raymond & Rousset 1995) are shown above diagonal, significant values indicated by ‘+’. Below diagonal are results of pairwise *F*_ST_, bold print marks significant (Bonferroni adjusted α-value = 0.00020) values. Letters indicate mountain regions according to Tab. 1.

|  | HE | FRA | SWA | EI | NCA | AFO | ML | JU | NAS | PIE | BK | CK | MFA | SLR | HU | PLA | DA | FCA | CA | PA | LA | APP | COR |
| --- | --- | --- | --- | --- | --- | --- | --- | --- | --- | --- | --- | --- | --- | --- | --- | --- | --- | --- | --- | --- | --- | --- | --- |
| HE |  | + | + | - | + | + | + | + | + | - | + | + | + | + | - | + | + | - | + | + | + | + | + |
| FRA | **0.198** |  | - | - | + | + | + | + | + | - | + | + | - | + | + | + | + | - | + | + | + | + | + |
| SWA | 0.262 | -0.028 |  | - | - | + | + | - | + | - | + | + | - | + | - | + | + | - | + | + | + | + | + |
| EI | -0.022 | 0.09 | 0.081 |  | + | + | + | + | + | - | + | + | - | + | - | + | + | - | + | + | + | + | + |
| NCA | **0.389** | **0.042** | 0.025 | **0.226** |  | + | + | + | + | - | + | + | - | + | + | + | + | + | + | + | + | + | + |
| AFO | **0.514** | **0.408** | **0.513** | **0.458** | **0.549** |  | + | + | + | - | + | + | + | + | + | + | + | + | + | + | + | + | + |
| ML | **0.314** | 0.136 | 0.171 | 0.22 | 0.193 | **0.32** |  | - | + | - | - | + | + | + | + | + | + | + | + | + | + | + | + |
| JU | **0.22** | **0.051** | 0.004 | **0.131** | 0.034 | **0.316** | -0.004 |  | + | - | + | + | - | + | + | + | + | + | + | + | + | + | + |
| NAS | 0.067 | **0.352** | **0.33** | **0.153** | **0.453** | **0.474** | **0.356** | **0.353** |  | + | + | + | + | + | + | + | + | + | + | + | + | + | + |
| PIE | 0.176 | -0.292 | -0.325 | -0.057 | -0.273 | 0.226 | -0.2 | -0.287 | 0.213 |  | - | + | - | - | - | - | - | - | + | - | - | + | + |
| BK | **0.712** | **0.582** | **0.866** | **0.656** | 0.777 | 0.642 | 0.526 | **0.467** | **0.566** | 1 |  | + | + | + | + | + | + | + | + | + | + | + | + |
| CK | **0.756** | **0.615** | **0.891** | **0.701** | **0.8** | **0.733** | **0.64** | **0.52** | **0.611** | 1 | 1 |  | + | + | + | + | + | + | + | + | + | + | + |
| MFA | 0.381 | -0.033 | -0.032 | 0.176 | -0.014 | **0.525** | 0.158 | -0.025 | **0.375** | 0 | 1 | **1** |  | + | + | + | + | - | + | + | + | + | + |
| SLR | **0.366** | **0.216** | 0.235 | **0.292** | **0.291** | **0.41** | 0.219 | **0.181** | **0.418** | -0.018 | **0.567** | **0.626** | 0.216 |  | + | + | + | + | + | + | + | + | + |
| HU | 0.026 | **0.106** | 0.07 | 0.005 | **0.202** | **0.326** | 0.13 | **0.119** | 0.134 | -0.164 | **0.472** | **0.459** | 0.109 | **0.231** |  | + | + | - | + | + | + | + | + |
| PLA | **0.459** | **0.58** | **0.539** | **0.486** | **0.634** | **0.521** | **0.481** | **0.508** | **0.426** | 0.39 | **0.586** | **0.641** | **0.54** | **0.551** | **0.422** |  | + | + | + | + | + | + | + |
| DA | **0.533** | **0.442** | **0.505** | **0.485** | **0.548** | **0.525** | **0.396** | **0.315** | **0.507** | 0.31 | **0.66** | **0.716** | **0.502** | **0.455** | **0.385** | **0.394** |  | + | + | + | + | + | + |
| FCA | 0.041 | 0.037 | -0.008 | 0.015 | **0.065** | 0.196 | 0.036 | **0.061** | **0.169** | -0.265 | 0.277 | 0.344 | -0.009 | 0.133 | 0.021 | **0.388** | **0.258** |  | + | + | + | + | + |
| CA | **0.785** | **0.845** | **0.789** | **0.799** | **0.846** | **0.75** | **0.74** | **0.813** | **0.788** | 0.695 | **0.737** | **0.774** | **0.774** | **0.794** | **0.759** | **0.764** | **0.789** | **0.735** |  | + | + | + | + |
| PA | **0.625** | **0.722** | **0.636** | **0.646** | **0.722** | **0.6** | **0.579** | **0.678** | **0.637** | 0.5 | **0.587** | **0.638** | **0.617** | **0.653** | **0.596** | **0.609** | **0.654** | **0.582** | **0.57** |  | + | + | + |
| LA | **0.55** | **0.675** | **0.545** | **0.577** | **0.642** | **0.5** | **0.481** | **0.625** | **0.592** | 0.383 | **0.457** | **0.518** | **0.519** | **0.578** | **0.532** | **0.552** | **0.565** | **0.582** | **0.328** | **0.355** |  | + | + |
| APP | **0.956** | **0.955** | **0.968** | **0.955** | **0.972** | **0.938** | **0.934** | **0.938** | **0.93** | 0.943 | 0.953 | **0.965** | **0.969** | **0.944** | **0.929** | **0.907** | **0.944** | **0.886** | **0.8** | **0.77** | **0.592** |  | + |
| COR | **0.982** | **0.984** | **0.985** | **0.982** | **0.989** | **0.973** | **0.971** | **0.977** | **0.975** | 0.972 | **0.976** | **0.982** | **0.985** | **0.978** | **0.972** | **0.966** | **0.978** | **0.96** | **0.932** | **0.93** | **0.814** | **0.975** |  |
