# Supplementary material for: From the Western Alps across Central Europe: Postglacial recolonisation of the tufa stream specialist Rhyacophila pubescens (Insecta, Trichoptera)
Source: Front Zool. 2011 May 15;8:10. doi: 10.1186/1742-9994-8-10 (PMC3119172; doi:10.1186/1742-9994-8-10)
Supplement: Additional file 2 — Regional differentiation based on AFLP data (FST). [file 1742-9994-8-10-S2.DOC]

Additional File, Engelhardt et al FIZ, EngelhardtEtAlFIZ_TableA2.doc

Table A2: Regional differentiation based on AFLP data. Below diagonal are results of pairwise F_ST_ based on AFLP data, bold print marks significant (Bonferroni adjusted α-value = 0.00020) values. Letters indicate mountain regions according to Tab. 1.

|  | HE | FRA | SWA | EI | NCA | AFO | ML | JU | NAS | PIE | BK | CK | MFA | SLR | HU | PLA | DA | FCA | CA | PA | LA | APP | COR |
| --- | --- | --- | --- | --- | --- | --- | --- | --- | --- | --- | --- | --- | --- | --- | --- | --- | --- | --- | --- | --- | --- | --- | --- |
| HE |  |  |  |  |  |  |  |  |  |  |  |  |  |  |  |  |  |  |  |  |  |  |  |
| FRA | **0.149** |  |  |  |  |  |  |  |  |  |  |  |  |  |  |  |  |  |  |  |  |  |  |
| SWA | 0.036 | 0.069 |  |  |  |  |  |  |  |  |  |  |  |  |  |  |  |  |  |  |  |  |  |
| EI | **0.312** | **0.386** | 0.379 |  |  |  |  |  |  |  |  |  |  |  |  |  |  |  |  |  |  |  |  |
| NCA | 0.102 | 0.083 | 0.172 | **0.312** |  |  |  |  |  |  |  |  |  |  |  |  |  |  |  |  |  |  |  |
| AFO | 0.05 | 0.253 | 0.115 | **0.452** | 0.253 |  |  |  |  |  |  |  |  |  |  |  |  |  |  |  |  |  |  |
| ML | 0.291 | **0.471** | 0.541 | 0.677 | **0.421** | 0.333 |  |  |  |  |  |  |  |  |  |  |  |  |  |  |  |  |  |
| JU | 0.148 | **0.194** | 0.139 | **0.327** | 0.125 | 0.151 | 0.291 |  |  |  |  |  |  |  |  |  |  |  |  |  |  |  |  |
| NAS | **0.276** | **0.237** | **0.358** | **0.481** | **0.157** | **0.373** | 0.249 | **0.211** |  |  |  |  |  |  |  |  |  |  |  |  |  |  |  |
| PIE | -0.007 | 0.235 | 0.266 | 0.537 | 0.067 | 0.233 | 0.547 | 0.033 | 0.071 |  |  |  |  |  |  |  |  |  |  |  |  |  |  |
| BK | 0.878 | 0.951 | 0.916 | 0.953 | **0.944** | 0.898 | 0.932 | **0.906** | 0.945 | 0.883 |  |  |  |  |  |  |  |  |  |  |  |  |  |
| CK | 0.51 | **0.717** | 0.473 | **0.661** | **0.704** | 0.426 | 0.478 | **0.638** | **0.703** | 0.304 | 0.111 |  |  |  |  |  |  |  |  |  |  |  |  |
| MFA | **0.317** | **0.508** | 0.594 | 0.149 | **0.369** | 0.569 | 0.871 | 0.319 | **0.545** | 0.791 | 0.957 | 0.543 |  |  |  |  |  |  |  |  |  |  |  |
| SLR | **0.25** | **0.397** | 0.468 | 0.07 | **0.243** | 0.464 | 0.746 | 0.26 | **0.441** | 0.574 | 0.951 | 0.56 | 0.045 |  |  |  |  |  |  |  |  |  |  |
| HU | **0.292** | **0.454** | **0.515** | 0.104 | **0.329** | 0.512 | **0.813** | **0.304** | **0.525** | 0.711 | 0.958 | 0.586 | -0.013 | 0.012 |  |  |  |  |  |  |  |  |  |
| PLA | 0.18 | 0.149 | **0.362** | **0.514** | 0.04 | 0.426 | 0.55 | **0.194** | **0.167** | 0.226 | **0.959** | **0.671** | **0.655** | **0.497** | **0.605** |  |  |  |  |  |  |  |  |
| DA | 0.126 | **0.352** | 0.289 | **0.55** | 0.214 | 0.35 | 0.494 | 0.133 | 0.24 | 0.074 | 0.907 | 0.426 | 0.722 | 0.588 | 0.682 | 0.366 |  |  |  |  |  |  |  |
| FCA | 0.093 | **0.21** | 0.1 | **0.275** | **0.166** | 0.137 | 0.152 | **0.178** | **0.197** | -0.08 | 0.804 | **0.528** | 0.259 | 0.217 | 0.266 | 0.157 | -0.038 |  |  |  |  |  |  |
| CA | **0.334** | **0.496** | **0.324** | **0.458** | **0.466** | **0.329** | **0.34** | **0.427** | **0.477** | 0.206 | 0.753 | **0.462** | **0.403** | **0.392** | **0.427** | **0.447** | 0.232 | 0.179 |  |  |  |  |  |
| PA | 0.223 | **0.351** | 0.199 | **0.37** | **0.311** | 0.227 | 0.255 | **0.278** | **0.333** | 0.066 | **0.815** | **0.525** | **0.33** | **0.302** | **0.345** | **0.306** | 0.107 | 0.1 | **0.221** |  |  |  |  |
| LA | **0.421** | **0.573** | 0.392 | **0.516** | **0.562** | 0.38 | 0.396 | **0.513** | **0.561** | 0.285 | 0.544 | **0.359** | 0.434 | **0.442** | **0.462** | **0.521** | 0.344 | **0.421** | **0.308** | **0.369** |  |  |  |
| APP | **0.882** | **0.941** | 0.92 | **0.944** | **0.933** | 0.912 | 0.934 | **0.894** | **0.933** | 0.912 | 0.922 | **0.667** | 0.951 | **0.945** | **0.951** | **0.95** | 0.912 | **0.769** | **0.682** | **0.764** | **0.471** |  |  |
| COR | **0.861** | **0.931** | 0.88 | **0.926** | **0.925** | 0.865 | 0.888 | **0.891** | **0.925** | 0.851 | 0.52 | **0.284** | 0.911 | **0.91** | **0.918** | **0.93** | 0.869 | **0.806** | **0.761** | **0.811** | **0.58** | **0.892** |  |
